# Evaluation by imaging methods of cochlear implant candidates: radiological and surgical correlation

**DOI:** 10.1016/S1808-8694(15)30574-7

**Published:** 2015-10-19

**Authors:** Luiz Rodolpho Pena Lima Júnior, Marina David Rocha, Priscilla Vargas Walsh, Camila André Antunes, Clara Maria Dias Ferreira Calhau

**Affiliations:** 1 Medical residency in Otorhinolaryngology; subspecialty in audiology. Specialized in cochlear implants. Doctoral student. ENT physician at the Clínica Otocento-RN, at the Centro SUVAG-RN, and at the Hospital do Coração de Natal.; 2 Medical student, 6th year, Universidade Federal do Rio Grande do Norte.; 3 Medical student, 6th year, Universidade Federal do Rio Grande do Norte.; 4 Medical student, 5th year, Universidade Federal do Rio Grande do Norte.; 5 Medical residency in Otorhinolaryngology. ENT physician at the Clínica Otocento-RN, at the Centro SUVAG-RN, and at the Hospital do Coração de Natal. Hospital do Coração de Natal, Clínica Otocentro-RN and Centro SUVAG-RN, Rio Grande do Norte.

**Keywords:** evaluation by imaging methods, cochlear implant, magnetic resonance imaging, deafness, computed tomography

## Abstract

Evaluation by imaging methods is critical in the preoperative care of cochlear implant (CI) surgery, providing safety to surgeons when indicating and performing this procedure. The ideal imaging study consists of an association between Computed Tomography (CT) and Magnetic Resonance Imaging (MRI).

**Aim:**

To investigate the accuracy of imaging studies as predictors of possible complications of surgery. Study Design: A cross-sectional investigation.

**Material and Method:**

The medical records of 104 patients undergoing CI surgery between May 2003 and October 2006 were studied. The preoperative muldisciplinary selection process included CT associated or not with MRI.

**Results:**

The final sample was composed of 100 patients after 4 patients with no records of radiological exams were excluded. Patients were divided into two groups. The accuracy of group A (CT only) was 69.69%, the sensitivity was 36.36%, the specificity was 86.36%, the Positive Predictive Value (PPV) was 57.14%, and the Negative Predictive Value (NPV) was 73.07%; the accuracy of group B (CT and MRI) was 80.59%, the sensitivity was 38.46%, the specificity was 90.74%, the PPV was 50.0%, and the NPV was 85.96%.

**Conclusion:**

The preoperative radiological evaluation by CI was effective in identifying anatomic abnormalities, allowing surgeons to avoid, or at least be aware of, possible complications. This study demonstrated that CT and MRI were superior to CT alone.

## INTRODUCTION

The cochlear implant (CI) is a highly technological device that is surgically inserted in the cochlea of patients with severe to profound bilateral sensorial dysacusis,^1^ and that have not benefited from conventional sound amplification hearing aids.^2^ The aim is to electrically stimulate auditory nerve fibers^3^ to partially replace cochlear function.

Candidates for the CI undergo preoperative assessment involving clinical, speech therapeutic, psychological and social criteria. During this stage, imaging of the cochlear region is paramount in defining the etiology of hearing loss, in locating findings that may contraindicate surgery, in helping to select the ear to be implanted, in adequately evaluating the anatomy for surgery, and - within limits - in predicting possible complications.

Given such importance, the ideal evaluation would include high resolution computed tomography (CT) associated with magnetic resonance imaging (MRI) of the temporal bone and the central nervous system (CNS);^4^, ^5^, ^6^ some authors go as far as recommending three-dimensional reconstruction of MRI images.^7^, ^8^ The cost of these exams, however, precludes their routine use for most of the patients at our unit. Thus, CT is always done; MRI is added when there are congenital cochlear deformities, a history of meningitis, hypoplasia of the inner auditory canal,^9^ congenital anatomic deformities of the temporal bone detected by CT, signs of inner ear ossification, temporal bone fracture, deafness due to otospongiosis, deafness due to auto-immune diseases, congenital deafness with anacusis, deafness due to syndromic diseases, and associated CNS involvement; a further reason when none of the above is present is when the patient has the means to pay for this exam. Adequate technique and an experienced radiologist are essential for assessing the local anatomy; structures may, thus, be adequately assessed, which enables surgeons to define the adequate approach for each case and to safely indicate CIs.

The purpose of this study was to assess the accuracy of image exams as predictors of possible complications during surgery, by correlating preoperative radiological signs with surgical findings.

## MATERIAL AND METHOD

### Sample selection

The study population was composed of patients of the Cochlear Implant Program (CIP) that underwent CI surgery in the city of Natal, Rio Grande do Norte (RN) state.

The CIP consists of three modules:

Module I diagnosis and selection.
Module II CI surgery.
Module III follow-up and (re)habilitation.

In module I, a multidisciplinary team of otorhinolaryngologists, speech therapists, social workers and psychologists evaluates the patients, which spend at least 3 months using bilateral conventional hearing aids. This stage aims to establish the etiology and diagnosis of their audiological condition, and to select patient for CI surgery.

Our sample consisted of 104 CIP patients who underwent CI surgery between May 2003 and October 2006. All patients were radiologically assessed by CT, with or with no MRI, and were operated by the same surgeon.

### Inclusion criteria

All patients that underwent CI surgery between May 2003 and October 2006 in the city of Natal, RN state, were included.

### Exclusion criteria

Patients with no records of preoperative exams or that refused to sign the free informed consent form that is part of the preoperative routine in our unit were excluded from this sample.

## METHOD

A cross-sectional, retrospective, comparative study was made of the charts of patients that underwent CI surgery to investigate variables such as sex, age at the time of surgery, origin, classification of deafness according to its etiology, onset and type of hearing loss, preoperative radiological exams and their findings, implanted ear, and intraoperative findings of each patient.

Image exams were done at different radiology units, and were not standardized.

Radiological and surgical findings were compared to judge the accuracy of the diagnostic tests. Compatible data were grouped, for statistical purposes, into true positive and true negative, and incompatible data were grouped into false positive (radiological findings not confirmed at surgery) and false negative (normal inner and middle ear anatomy not confirmed at surgery).

The Statistica software was used for analyzing the variables. Tables were drawn on Microsoft Excel® 2003 and charts were drawn on Microsoft Excel® 2003 and the HG® software.

The Research Ethics Committee of the Universidade Federal do Rio Grande do Norte approved this study (protocol number CEP/UFRN-078/06).

## RESULTS

**Chart 1:**
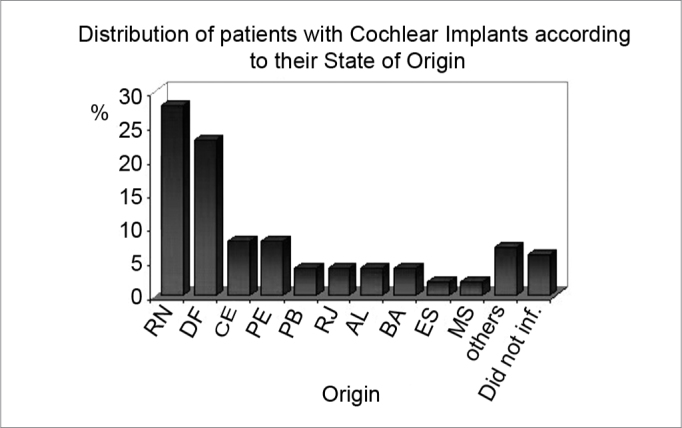
Distribution of patients with Cochlear Implants according to their State of Origin.

The original sample consisted of 104 patients. Four patients, however, were excluded; although they had done preoperative image exams, the results were not recorded in their charts and/or patients did not send the exams clinical unit. The final sample, therefore, consisted of 100 patients, of whom 59% were male and 85% were children aged below 10 years (WHO criterion). The origin of patients may be seen on Chart 1.

**Chart 2:**
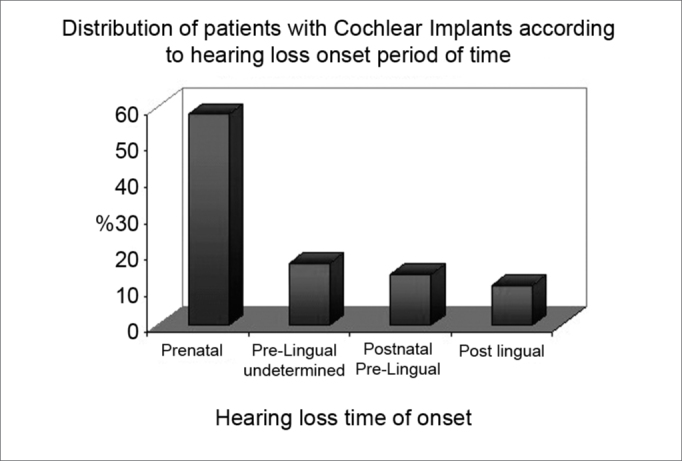
Distribution of patients with Cochlear Implants according to hearing loss onset period of time

**Chart 3:**
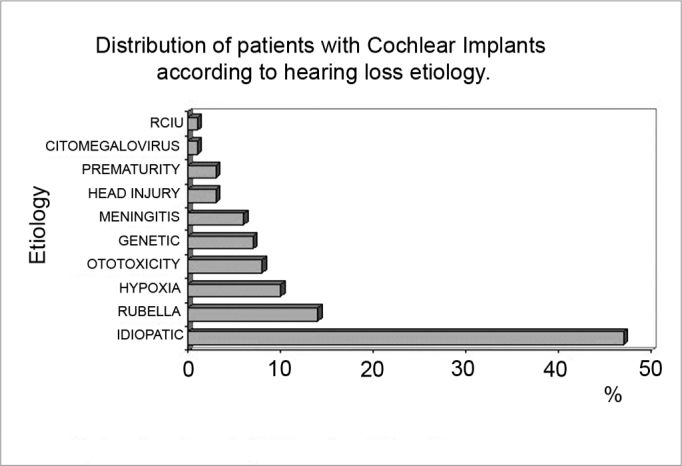
Distribution of patients with Cochlear Implants according to hearing loss etiology.

All patients had severe to profound bilateral deafness, of which 58% had prenatal deafness, 17% had undefined prelingual deafness, 14% had postnatal prelingual deafness, and 11% had postlingual deafness (Chart 2). The etiology of deafness was as follows: 47% were idiopathic, 14% due to congenital rubella, 10% due to perinatal hypoxia, 8% due to ototoxicity, 7% were hereditary (5 non-syndromic and 2 syndromic cases), 6% due to meningitis, 3% due to prematurity, 3% due to cranial trauma, 1% due to cytomegalovirus and 1% due to restricted intrauterine growth (Chart 3).

The preoperative radiological investigation for CI surgery was as follows: all patients (100%) underwent CT; 67% of patients also underwent MRI. This resulted in two groups, those that only underwent CT (33 patients) and those that underwent CT and MRI (67 patients).

### Radiological findings

Radiological exams revealed that 30 of 100 patients had alterations such as inflammation, a high jugular bulb, widening of the vestibular aqueduct, ossification, and congenital malformation, among other findings.

As to the type of exam that disclosed alterations, 20 patients had alterations only on CT (although 5 of these underwent CT and MRI), 2 had alterations only on MRI (although these patients also underwent both exams), and 8 had alterations on both CT and MRI (Table 1).

**Table 1:** Distribution of implanted patients according to image exam and findings

FINDINGS	TYPE OF EXAM					
	CT	%	MRI	%	CT + MRI	%
Inflammation	12	60,0	1	50,0	5	62,5
Widening of the vestibular aqueduct	1	5,0	0	0,0	1	12,5
Vascular changes	3	15,0	0	0,0	0	0,0
Ossification	0	0,0	1	50,0	0	0,0
Malformation	1	5,0	0	0,0	0	25,0
Others	3	15,0	0	0,0	2	0,0

Total	20	100,0	2	100,0	8	100,0

Computed Tomography.

### Surgical findings

Surgery showed intraoperatory findings in 23 of 100 patient; 10 had adhesions, 4 had an effusion in the auditory cleft, 4 had ossification, 3 had a high jugular bulb, 1 had a gusher and 1 had malformation + gusher.

### Radiological X surgical findings

Patients in which the ear alteration was unilateral, and in whom CI surgery was done in the contralateral (normal) ear, were considered as having no radiological alterations for the purpose of comparing radiological and surgical data. Thus, there were 16 patients with and 84 patients without altered radiological exams.

The comparison of radiological and surgical findings was done separately for both groups. GROUP A consisted of patients that had undergone CT only; GROUP B consisted of patients that had undergone CT and MRI.

### GROUP A

In 33 patients, radiological findings were similar to surgical findings in 23 cases (69.69% accuracy). Of these, 19 patients had no radiological abnormalities, which was confirmed during surgery, 2 patients had a high jugular bulb that was confirmed by surgery, 1 patient had widening of the vestibular aqueduct and 1 patient had inflammation, both confirmed surgically by the presence of a gusher in the former and adhesions in the latter. Of the remaining 10 patients, 3 had radiological evidence of inflammation that was not confirmed by surgery, and 7 patients with normal exams had alterations that were found during surgery (3 had effusion of the auditory cleft, 3 had adhesions and 1 had ossification) (Table 2). The sensitivity was 36.36%, the specificity was 86.36%, the positive predictive value was (PPV) was 57.14%, and the negative predictive value (NPV) was 73.07%.

**Table 2:** Distribution of ci patients (group a) according to the correlation between surgery and CT

SURGICAL FINDINGS	CT FINDINGS								
	I	%	WVA	%	Vascular	%	No	%	Total
Adhesions	1	25,0	0	0,0	0	0,0	3	11,5	4
HJB	0	0,0	0	0,0	2	100,0	0	0,0	2
EAC	0	0,0	0	0,0	0	0,0	3	11,5	3
Ossification	0	0,0	0	0,0	0	0,0	1	3,8	1
Gusher	0	0,0	1	0,0	0	0,0	0	0,0	1
No	3	75,0	0	0,0	0	0,0	19	73,1	22

Total	4	100,0	1	0,0	2	100,0	26	100,0	33

### GROUP B

In 67 patients, radiological findings were similar to surgical findings in 54 cases (80.59% accuracy). Of these, 49 patients had no radiological abnormalities (confirmed during surgery), 3 patients had inflammation on image studies (in 1 case it was seen only on CT, and in 2 patients it was seen on both exams) that was confirmed during surgery (2 patients with adhesions and 1 patient with effusion of the auditory cleft). In 1 case, widening of the vestibular aqueduct was confirmed by the finding of a gusher during surgery. A high jugular bulb was seen in 1 patient, which was confirmed by surgery. There were 13 patients whose results were not in agreement. Of these, radiology was normal in 8 patients that had positive surgical findings (4 had adhesions, 3 had ossification, 1 had a malformation + a gusher). The other 5 patients had radiological findings that were not confirmed during surgery; 3 patients had radiological signs of inflammation (1 only on CT and 2 in both CT and MRI), 1 patient had ossification seen on MRI, and 1 patient had thickening of the basal turn (on CT) (Table 3). The sensitivity was 38.46%, the specificity was 90.74%, the PPV was 50% and the NPV was 85.96%.

**Table 3:** Distribution of ci patients (group b) according to the correlation between surgical and radiological findings (CT + MRI)

SURGICAL FINDINGS	CT AND/OR MRI FINDINGS												
	I	%	Ossification	%	HJB	%	WVA	%	Other	%	No	%	Total
Ossification	0	0,0	0	0,0	0	0,0	0	0,0	0	0,0	3	5,3	3
Adhesions	2	33,3	0	0,0	0	0,0	0	0,0	0	0,0	4	7,0	6
HJB	0	0,0	0	0,0	1	100,0	0	0,0	0	0,0	0	0,0	1
EAC	1	16,7	0	0,0	0	0,0	0	0,0	0	0,0	0	0,0	1
Gusher	0	0,0	0	0,0	0	0,0	1	100,0	0	0,0	0	0,0	1
Malformation and Gusher	0	0,0	0	0,0	0	0,0	0	0,0	0	0,0	1	1,8	1
No	3	50,0	1	100,0	0	0,0	0	0,0	1	100,0	49	86,0	54

Total	6	100,0	1	100,0	1	100,0	1	100,0	1	100,0	57	100,0	67

## DISCUSSION

Temporal bone images prior to placing a CI are useful for checking the local anatomy and pathology to identify factors that may interfere with surgery or implant function.^10^ Image studies are, thus, essential before CI surgery. Structural alterations of the cochlea, the middle ear and the mastoid should be identified to help guide the surgical procedure.^9^

CT and RNM provide different, but complementary information.^5^ CT is excellent for demonstrating details of the temporal bone,^11^, ^12^ mastoid pneumatization,^4^, ^9^ and cochlear patency.^13^, ^14^ CT is inadequate for visualizing inner ear neural structures, fluid or fibrosis.^5^, ^14^, ^15^, ^16^ MRI is superior to CT in demonstrating inner auditory canal nerves, retrocochlear diseases and membranous alterations of the inner ear; it fails to provide information about bone structures,^5^, ^9^, ^14^, ^15^ and is more costly.^5^

As has been established in the literature, preoperative radiology is expected to support the indication of CI surgery and the choice of ear to be implanted in any given patient.

Regardless of the type of exam (CT or CT + MRI), both were useful for assuring normal inner ear anatomy (CT: specificity - 86.36%, NPV - 73.07%; CT + MRI: specificity - 90.74%, NPV - 85.96%), which supported adequate patient selection for CI surgery.

Imaging, however, had a poor performance in detecting abnormalities (CT: sensitivity - 36.36%; CT + MRI: sensitivity - 38.46%) and in assuring the reliability of results (CT: PPV - 57.14%; CT + MRI: PPV - 50,0%).

There were eight radiological alterations that were not confirmed by surgery, seven of which were inflammation, and 15 surgical findings that had not been identified by the image exams, 10 of which were also inflammation. These results may be debated and optimized if we consider that inflammation may have started or resolved during the time interval between the image exam and CI surgery.^9^ At our unit, otoscopy and tympanometry are done periodically and 24 hours before surgery to monitor those patients that have inflammation as a radiological finding; this aims to verify that this condition has resolved, so that surgical intercurrences may be minimized. The presence of a high jugular bulb - if visualized preoperatively - does not contraindicate CI surgery.^9^ Ossification contraindicates CI surgery if the resulting obstruction does not allow electrodes to be placed.^9^ However, this also depends on the experience of the surgeon, and may only prolong surgical time,16 as in the sample cases.

A correlation between the results of both groups shows that when CT and MRI are done, there is increased agreement with surgical compared to only CT (CT: accuracy - 69.69%; CT + MRI: accuracy - 80.59%).

Our data suggests that image exams were useful in supporting the choice of ear for the CI - avoiding unilateral conditions - in 14% of cases. In these cases, however, the agreement between imaging methods and surgery was only 57.1% (8 of 14); in the remaining 6 cases, there were positive surgical findings when radiology had described lack of abnormalities. Again, as in the aforementioned discussion about inflammation, which might have arisen in the interval between the exam and surgery due to its high rate among surgical findings (5 of 6 cases), the radiological findings about unilateral conditions may not necessarily be an error, since there is no assurance that an abnormality found during surgery would not also be present in the contralateral ear.

Furthermore, abnormalities detected during surgery in the ear selected for CI had less influence than the detection of alterations in the discarded ear would have had (decreased cochlear patency, shortened semicircular canal and congenital malformation).

Knowing that the exam technique may have a significant influence on the results, we believe that standardization based on established criteria are needed for assessing anatomical structures for surgery, and that this would optimize the predictive ability of these exams, increasing the radiological and surgical agreement.

## CONCLUSION

Preoperative imaging before CI surgery, notwithstanding its limitations, is important, particularly when done according to ideal standards. This approach supports not only selecting appropriate cases for CI surgery, but also preparing surgeons for overcoming abnormalities and avoiding complications that could potentially have a negative effect on the procedure and its results. Full electrode insertion and the absence of complications related to preoperative radiological findings or absence of findings was reported in all patients. Complications include: perilymphatic leak, absence of electrical stimulation of the VIII nerve, facial paresis or palsy, facial stimulation, and postoperative meningitis. Similar to well documented opinions in the literature, the current study demonstrates that preoperative CT and MRI are more accurate that CT singly.
